# A Novel Immune-Related Prognostic Model for Response to Immunotherapy and Survival in Patients With Lung Adenocarcinoma

**DOI:** 10.3389/fcell.2021.651406

**Published:** 2021-03-19

**Authors:** Yujia Zheng, He Tian, Zheng Zhou, Chu Xiao, Hengchang Liu, Yu Liu, Liyu Wang, Tao Fan, Bo Zheng, Fengwei Tan, Qi Xue, Gengshu Gao, Chunxiang Li, Jie He

**Affiliations:** ^1^Department of Thoracic Surgery, National Cancer Center/National Clinical Research Center for Cancer/Cancer Hospital, Peking Union Medical College, Chinese Academy of Medical Sciences, Beijing, China; ^2^Department of Colorectal Surgery, National Cancer Center/National Clinical Research Center for Cancer/Cancer Hospital, Peking Union Medical College, Chinese Academy of Medical Sciences, Beijing, China; ^3^Department of Pathology, National Cancer Center/National Clinical Research Center for Cancer/Cancer Hospital, Peking Union Medical College, Chinese Academy of Medical Sciences, Beijing, China

**Keywords:** lung adenocarcinoma, immune infiltration, prognosis, immunotherapy, risk prediction model, signature

## Abstract

Lung adenocarcinoma is one of the most malignant diseases worldwide. The immune checkpoint inhibitors targeting programmed cell death protein 1 (PD-1) and programmed cell death-ligand 1 (PD-L1) have changed the paradigm of lung cancer treatment; however, there are still patients who are resistant. Further exploration of the immune infiltration status of lung adenocarcinoma (LUAD) is necessary for better clinical management. In our study, the CIBERSORT method was used to calculate the infiltration status of 22 immune cells in LUAD patients from The Cancer Genome Atlas (TCGA). We clustered LUAD based on immune infiltration status by consensus clustering. The differentially expressed genes (DEGs) between cold and hot tumor group were identified. Gene Ontology (GO) and Kyoto Encyclopedia of Genes and Genomes (KEGG) enrichment analysis were performed. Last, we constructed a Cox regression model. We found that the infiltration of M0 macrophage cells and follicular helper T cells predicted an unfavorable overall survival of patients. Consensus clustering of 22 immune cells identified 5 clusters with different patterns of immune cells infiltration, stromal cells infiltration, and tumor purity. Based on the immune scores, we classified these five clusters into hot and cold tumors, which are different in transcription profiles. Hot tumors are enriched in cytokine–cytokine receptor interaction, while cold tumors are enriched in metabolic pathways. Based on the hub genes and prognostic-related genes, we developed a Cox regression model to predict the overall survival of patients with LUAD and validated in other three datasets. In conclusion, we developed an immune-related signature that can predict the prognosis of patients, which might facilitate the clinical application of immunotherapy in LUAD.

## Introduction

Lung cancer is the most common fatal disease in the world, causing most cancer-related death every year. Eighty-five percent of lung cancer are non-small cell lung cancer (NSCLC) (Siegel et al., 2020). As the most frequently diagnosed subtype of NSCLC, lung adenocarcinoma (LUAD) has high inter-/intratumor heterogeneity, and its carcinogenic mechanisms have not been fully illustrated (Calvayrac et al., 2017). Before the introduction of immunotherapy, the outcomes of LUAD patients were dismal due to its malignant nature and limited effect of chemotherapy. However, with the rapid development of immune checkpoint inhibitors and target therapy, the prognosis of patients has improved significantly (Herbst et al., 2018). To diagnose and treat patients more precisely and economically, effective and stable models that can predict and stratify the prognosis of LUAD patients is warranted (Tang et al., 2017).

The introduction of immunotherapy revolutionized the paradigm of cancer treatment, and it brought hope to patients who were formerly untreatable and improved the survival status of many LUAD patients (Doroshow et al., 2019). However, there are still some patients who are resistant to and cannot benefit from immune check point blocker (ICB). Among patients who are resistant to ICB, some of them do not respond to immunotherapy (innate resistance), and others initially respond to ICB but turned to be insensitive as the disease progresses (acquired resistance) (Pitt et al., 2016). One of the main mechanisms underlying the immunotherapy resistance is immune evasion, which is utilized by tumor cells to escape the immune surveillance and elimination (Vinay et al., 2015; Herbst et al., 2018). Under this aberrant situation, immune responses aroused by the tumor antigen can be suppressed in tumor microenvironment (TME), which is dynamic and complex, consisting of several immune cells, stromal cells, cytokines and chemokines, and extracellular molecules, and immune cell infiltration status is the key determinant of TME (Altorki et al., 2019). Like a double-edged sword, TME is able to lead to both beneficial and adverse consequences in tumorigenesis, and TME can change continually in the process of tumor progression (Quail and Joyce, 2013).

The development of next-generation sequencing enabled us to characterize tumor heterogeneity from the gene level, and the public databases such as TCGA provide us with a chance to guide and design basic experiments (Devarakonda et al., 2015). Using bioinformatic technology, we can analyze the immune infiltration in tumors and calculate the value of the immune/stromal score for LUAD.

In our study, we calculate 22 immune cells in LUAD and identified 5 clusters of LUAD based on the infiltration status of immune cells. To further explore the mechanism behind the infiltration of immune cells, we defined two groups, cold tumor and hot tumor, based on the five clusters. We developed a Cox regression model based on the DEGs and made validation in three external cohorts. Overall, we are the first to classify patients in LUAD based on immune cell infiltration retrieved from TCGA and construct a stable predicting model for survival of LUAD patients. Our findings may give guidance to the application of immunotherapy and facilitate the clinical management of LUAD.

## Materials and Methods

### Data Collection

The RNA-sequencing data and clinical information of LUAD were downloaded from UCSC XENA (http://xena.ucsc.edu/). The RNA-sequencing data for LUAD with immunotherapy were downloaded from the platform supplied in the articles (Hugo et al., 2016; Riaz et al., 2017; Mariathasan et al., 2018). The processed count data of the bladder cancer were download from an online website (http://research-pub.gene.com/IMvigor210CoreBiologies/) supplied in the article. The processed count and fragments per kilobase of transcript per million mapped reads (FPKM) data were downloaded from the Gene Expression Omnibus (GEO) database (https://www.ncbi.nlm.nih.gov/) with accession numbers: GSE78220 and GSE91061. These three data sets were transformed into transcripts per million (TPM) and made a log(x + 1) normalization.

### Immune Infiltration Estimation

The estimation of 22 immune cells of LUAD was calculated by R package “CIBERSORT;” the samples with *p* < 0.05 were included for further analysis (Newman et al., 2015; Thorsson et al., 2018). The 28 immune cells were calculated by R package “ssGSEA” with supplied cell makers (Tamborero et al., 2018). The six-cell types were accessed from an online tool TIMER (https://cistrome.shinyapps.io/timer/) (Li et al., 2020). The R package “ESTIMATE” was applied to calculate the immune score, stromal score, and tumor purity. To explore the immune infiltration in LUAD, we used CIBERSORT to calculate the proportion of 22 immune cells and revealed the function of immune infiltration through multiple strategies.

### Differentially Expressed Genes Selection

The samples were divided into two main groups based on the immune score and immune cell infiltration. The R package “limma” was used to calculate DEGs with criteria as follows: logFC > 1 or < -1 and adjust *p* < 0.05. Visualization of DEGs was conducted by volcano diagram and heatmap.

### Enrichment and Protein–Protein Network Analysis

For the enrichment analysis, genes with *p* < 0.05 that were differentially expressed in hot and cold tumors were selected. We use the R package “clusterprofile” to perform GO enrichment categories. The Database for Annotation, Visualization, and Integrated Discovery (DAVID, https://david.ncifcrf.gov/), an online tool, was used to perform KEGG pathway analysis. STRING (https://string-db.org/) was used to conduct a protein–protein interaction (PPI) network and further visualized by Cytoscape.

### Consensus Clustering

The consensus clustering of 22 immune cells was performed by the R packages “ConsensusClusterPlus” with reps = 100, pItem = 0.8, and pfeature = 1. The optimal number of clusters is determined by heat map and delta diagram.

### Construction of Predicting Model

The LUAD RNA-sequencing data with survival information were randomly divided into training and testing cohort by R package “caret.” Genes differently expressed in hot and cold tumors were used to perform univariate survival analysis, and genes with *p* < 0.05 were selected. Then, the R packages “glmnet” was used to perform least absolute shrinkage and selection operator (LASSO) analysis. To optimize the model, a step-wised proportional hazards model was used. The survival analysis was analyzed by R packages “survival,” and receiver operating characteristic (ROC) was analyzed by R package “survivalROC.”

### Statistical Analysis

All analyses used in this study were performed by R software (version 3.5.1). For the analysis of the correlation of immune infiltration and clinical–pathological parameters, cells were divided into two groups based on the clinical parameters, and chi-square test was used to analyze the correlation. WilcoxTest was used to compare the infiltration of immune cells in normal and tumor tissues, as well as in cold and hot tumors. ANOVA was used to compare immune score, stromal score, and tumor purity among the five clusters. For the survival analysis, *p*-value was calculated with log-rank test. *p* < 0.05 was considered as statistically significant.

## Results

### Correlation of Immune Infiltration and Clinical Parameters in LUAD

**Figure 1:**
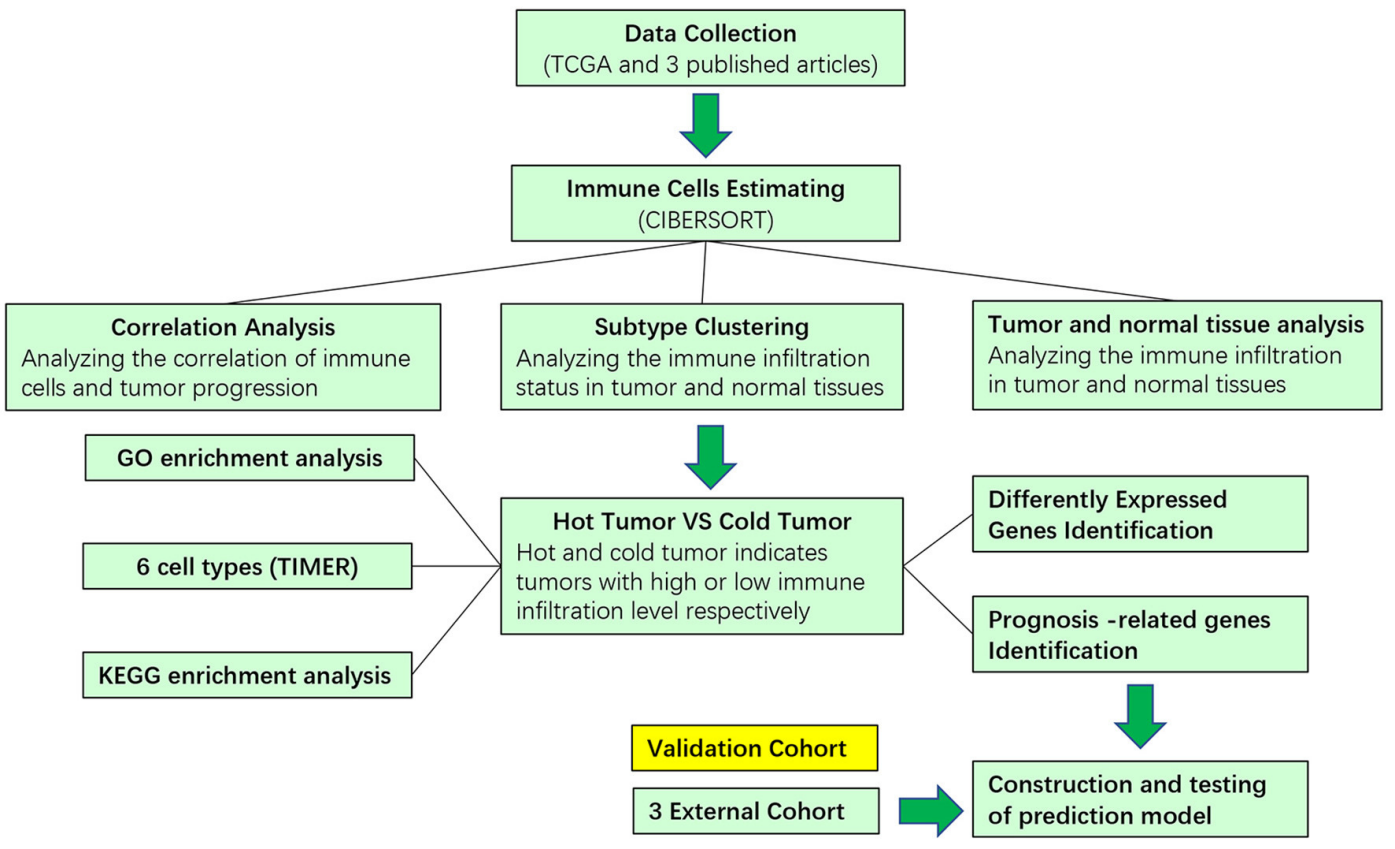
Schematic flowchart showed the analysis strategy.

**Figure 2:**
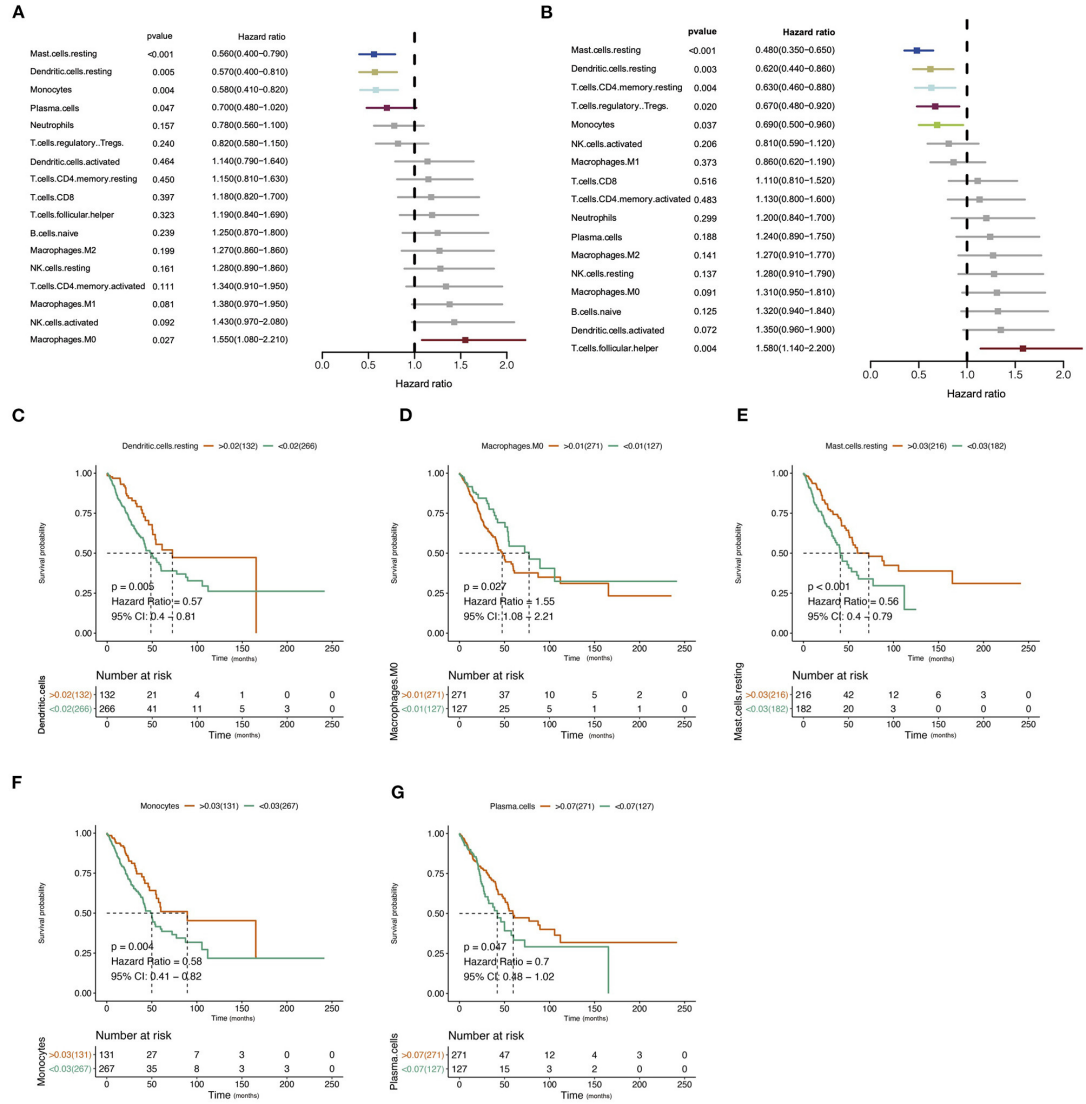
Correlation of immune infiltration and clinical parameters in lung adenocarcinoma (LUAD). **(A)** Forest plot showed the correlation of immune infiltration and overall survival (OS). **(B)** Forest plot showed the correlation of immune infiltration progression-free interval (PFI). **(C–G)** The Kaplan–Meier diagram showed the correlation of infiltration of immune cells and overall survival (OS).

The design and process of our study are shown in the flow chart in Figure 1. Survival analysis revealed that higher infiltration of dendritic cells, mast cells, monocytes, and plasma cells was associated with better overall survival of patients, while macrophages predicted an unfavorable outcome. In terms of progression-free interval (PFI), higher infiltration of dendritic cell, mast cell, monocyte, CD4^+^ T cell, and regulatory T cell (Tregs) were significantly correlated with longer survival, while follicular helper T cell points to negative prognosis. These results indicate that immune cells status can reflect tumor features, and the infiltration of immune cells has prognosis predicting function (Figure 2).

### Different Immune Cell Infiltration Patterns in Normal and Tumor Tissues

**Figure 3:**
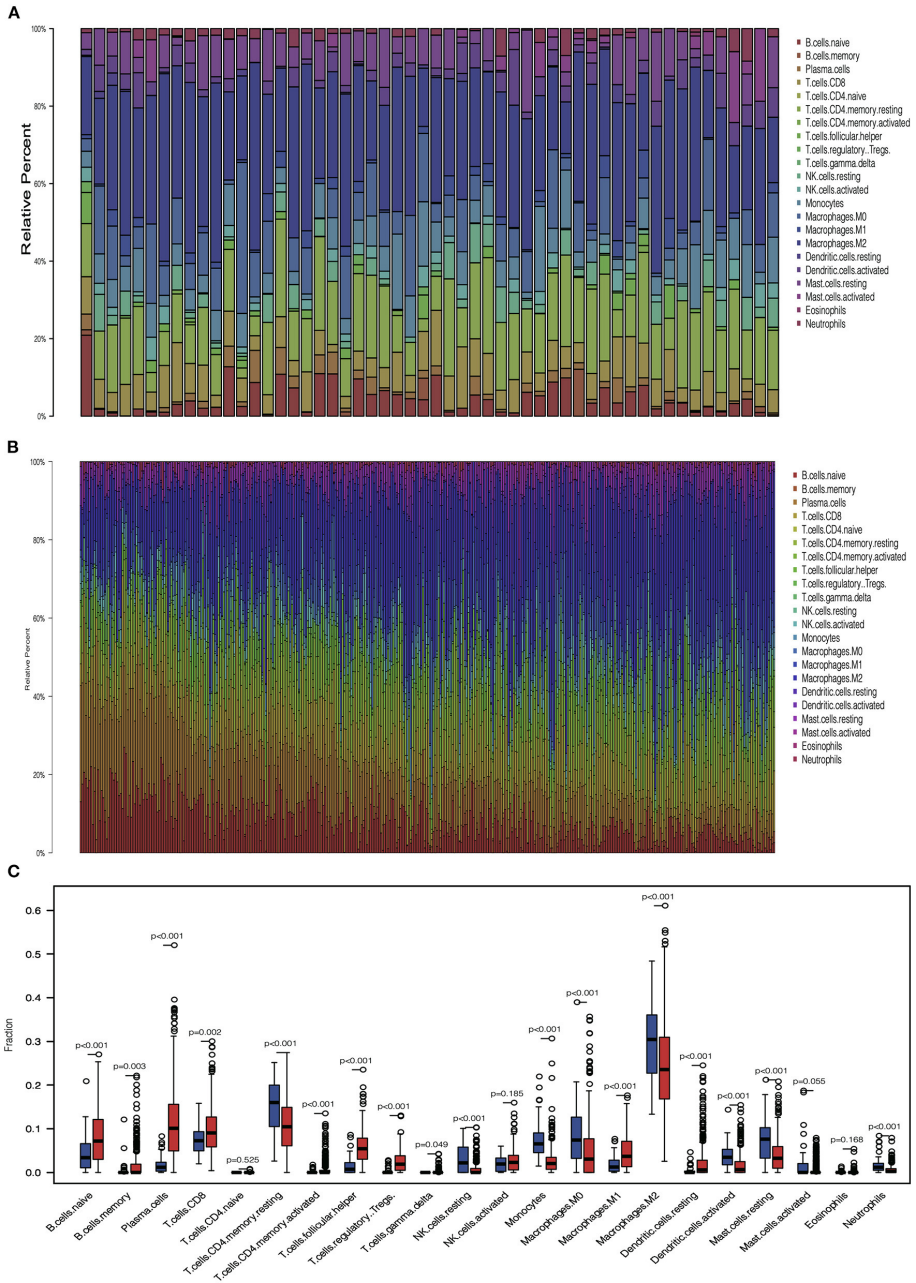
Immune cell infiltration pattern in tumor and normal tissue. **(A)** Barplot showed the distribution of 22 immune cells in normal tissue. **(B)** Barplot showed the distribution of 22 immune cells in tumor tissue. **(C)** Boxplot showed the 22 immune cells infiltration in normal and tumor tissue.

We analyzed the proportion of immune cells in tumors and normal tissues, respectively, to explore the infiltration of immune cells. Heterogeneity of LUAD was shown by the different ratios of each cell type (Figures 3A,B). Then, we compared the infiltration of immune cells in tumors and normal tissues. Results showed that several cells involved with tumor immunity have higher immune infiltration level in tumor tissues, including naive B cell, memory B cell, plasma cell, CD8^+^ T cell, activated CD4^+^ memory T cell, follicular helper T cell, regulatory T cell, M1 macrophage cell, and resting dendritic cell. In contrast, some types of cells in the resting status are abundant in normal tissues, including natural killer (NK) resting cell, mast resting cell, and resting CD4^+^ memory T cell. These results indicate that most immune cells accumulated in tumor tissues are in response to the tumor neoantigen (Figure 3C).

### Correlation of Immune Cells in Tumors and Normal Tissues

Cancer immune interaction is a process that involves multiple cell types, so it is important to characterize the synergistic or antagonistic relationships between different cells. Therefore, we performed a correlation analysis of the 22 immune cells in tumors and normal tissues. In tumor samples, we found that CD8^+^ T cells were positively associated with activated memory CD4^+^ T cell, follicular helper T cell, and M1 macrophage cells, indicating the cooperation among these cells. On the contrary, results showed that CD8^+^ T cells were negatively correlated with M2 macrophage cells in tumor samples, indicating that M1 and M2 macrophage cells exhibit different ability in regulating immune responses mediated by CD8^+^ T cells. Generally, immune cells showed much weaker correlation in normal samples compared with tumor samples. In normal samples, we found that naive B cells were positively correlated with regulatory T cells, plasma cells, and regulatory T cells, while follicular helper T cells were negatively associated with resting NK cells and activated dendritic cells.

**Figure 4:**
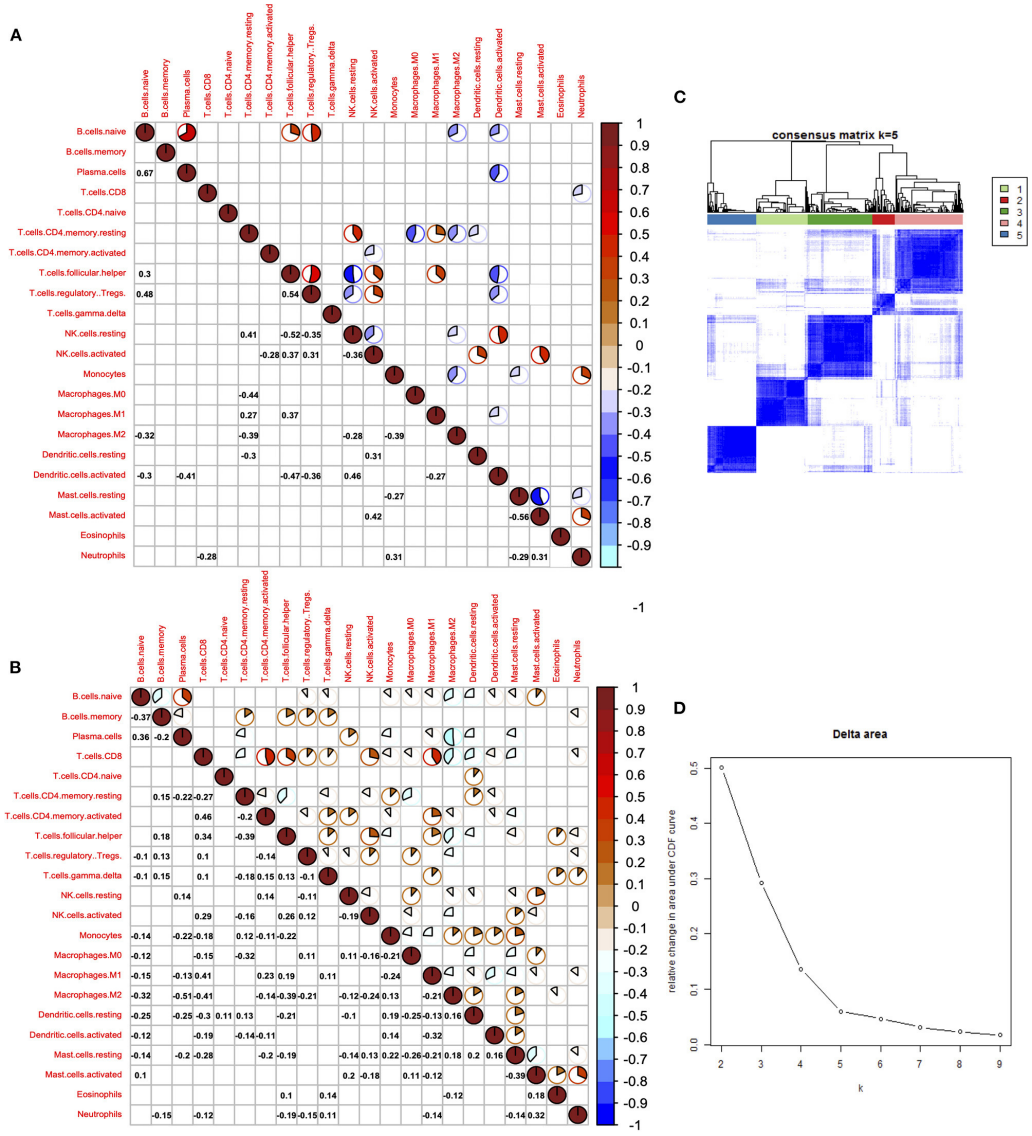
Correlation of immune cells in the tumor and normal tissues. **(A)** Corrplot showed the correlation of 22 immune cells in tumor tissues. **(B)** Corrplot showed the correlation of 22 immune cells in normal tissues. **(C)** Heatmap showed the clusters of immune cells. **(D)** Delta diagram showed the clusters with under area.

Among all 22 immune cell types, CD8^+^ T cells are the cell type that has diverse relationships with other cell types, indicating its pivotal role in immune regulation of LUAD. Positive correlations were also found between naive B cells and plasma B cells; mast cells and neutrophil were also positively correlated, suggesting a synergistic relationship between them (Figures 4A,B). Delta results and heatmap revealed that the LUAD could be divided into five clusters according to the different immune infiltration patterns (Figures 4C,D).

### Immune Subtyping of LUAD

**Figure 5:**
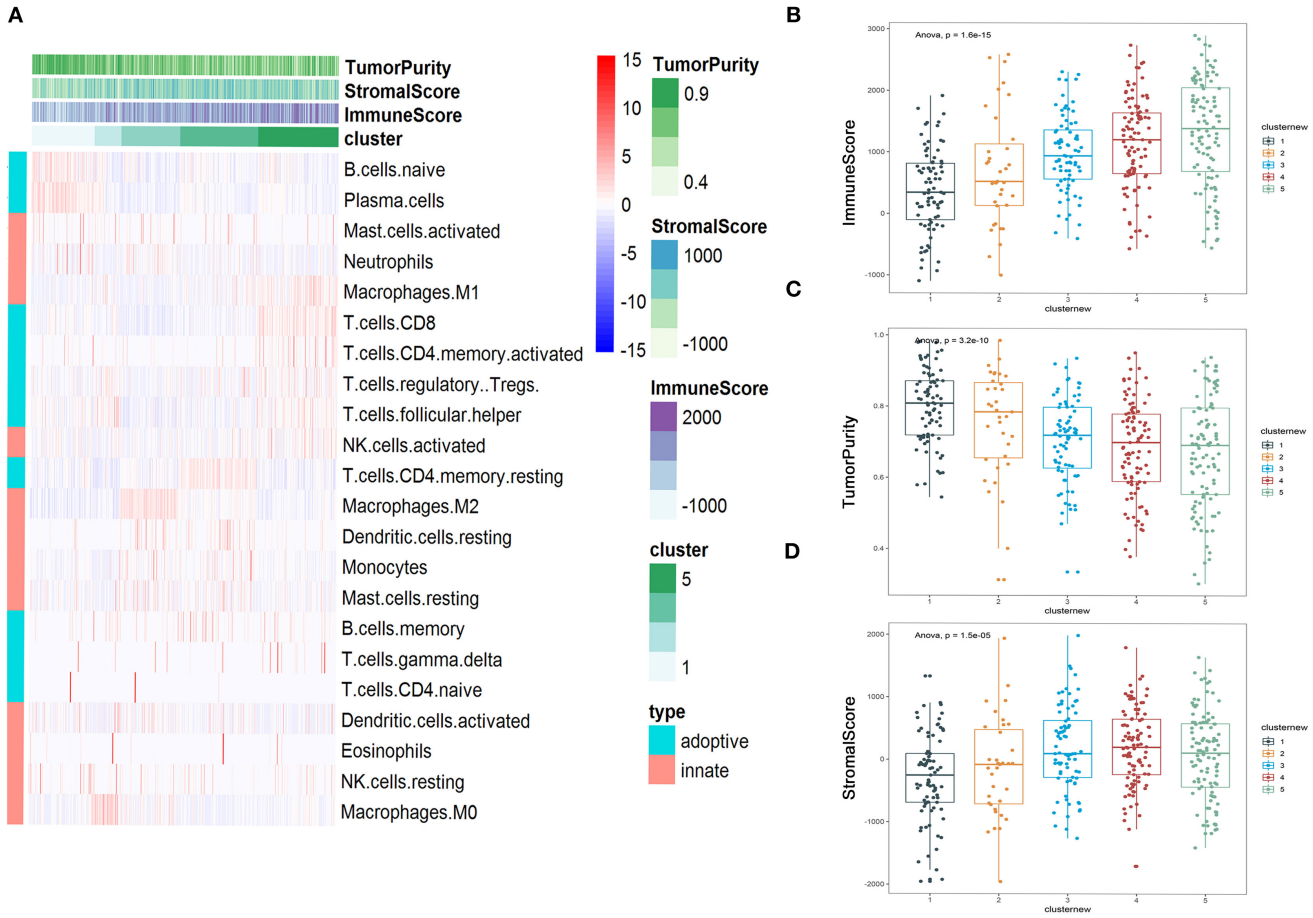
Immune subtyping of lung adenocarcinoma (LUAD). **(A)** Heatmap showed the immune clusters of LUAD. **(B–D)** Expression of the immune score, stromal score, and tumor purity in the five clusters.

Heatmap was performed to show the distribution of 22 immune cells in the 5 clusters in LUAD. Cluster 1 was mainly enriched in adopted immune cells, which were naive B cells and plasma cells. Cluster 2 was highly enriched in M0 macrophages of innate immune and activated NK cell and follicular helper T cell of adopted immune. Cluster 3 was strongly enriched in M2 macrophage and moderately enriched in neutrophils, resting dendritic cells, and resting mast cells. Cluster 4 was highly enriched in resting memory CD4^+^ T cells. Cluster 5 was mainly enriched in several kinds of T cell, including CD8^+^ T cell, activated memory CD4^+^ T cell, Tregs, follicular helper T cells, and two cell types from innate immune, which are M1 macrophages and activated NK cells, suggesting that innate immune and adopted immune may have a synergistic effect in the immune interaction (Figure 5A). The immune score, stromal score, and tumor purity were calculated to further profile the five clusters. Consistent with heatmap, cluster 5 had the highest immune score among all five clusters; while it had the lowest tumor purity, its stromal score was lower than that of cluster 4. The immune scores of the five clusters increased from cluster 1 to 5; consistent with this, the tumor purity of the five clusters decreased from cluster 1 to 5 gradually. The same tendency can be found in the stromal score, but cluster 4 had the highest stromal score (Figures 5B–D).

### Differences in Hot and Cold Tumor

**Figure 6:**
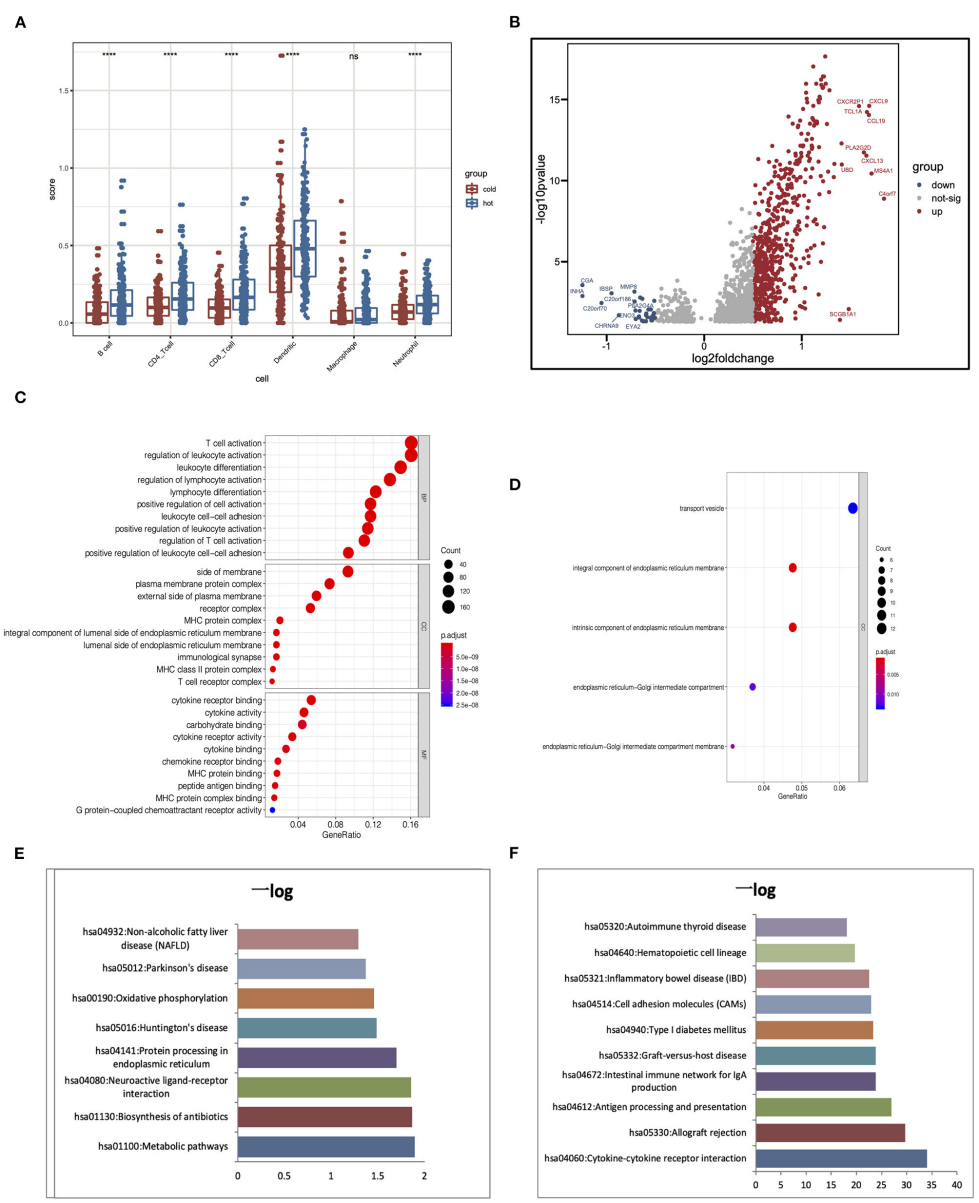
Alterations of signaling in hot and cold tumors. **(A)** Immune infiltration in hot and cold tumors analyzed by TIMER. **(B)** Differentially expressed genes between hot and cold tumors. **(C,D)** Gene Ontology (GO) enrichment analysis in hot and cold tumors. **(E,F)** Kyoto Encyclopedia of Genes and Genomes (KEGG) analysis in hot and cold tumors. ****p < 0.001; ns, not significant.

Hot tumors indicate tumors that have high immune infiltration; accumulating evidence has suggested that patients with hot tumors are more likely to benefit from immunotherapy. On the contrary, cold tumor with a low level of immune infiltration is prone to be resistant to ICB (Jiang et al., 2018; Mariathasan et al., 2018). Results showed that clusters 4 and 5 had higher immune scores than other clusters; although there are several immune cells enriched in clusters 1–3, they lacked the enrichment of CD8^+^ T cells, which was an important cell type related to immune therapy response. To further elaborate the mechanism that dictates the immune cell infiltration in tumors, we divided the five clusters of LUAD into two major groups: clusters 1–3 were cold tumors; clusters 4 and 5 were hot tumors. First, we estimate and compare immune cell infiltration levels in the cold and hot tumor groups. Both methods showed that compared to cold tumors, antigen-presenting cells and other important immune cells are highly infiltrated in hot tumors, which further approve our definition of LUAD (Figure 6A). Then, we explored the difference between the two groups at the transcriptional level. Volcano and heatmap showed that hot and cold tumors are different in transcription patterns (Figure 6B). Consistent with all above the results, immune-related genes were highly expressed in hot tumor, for instance, CXCL9, TCL1A, CCL19, CXCL13, MS4A1, and C4orf7. Although few immune-related genes had a high expression in cold tumor, such as MMP8, most genes highly express in cold tumor are not so closely related to immune responses, including CGA, INHA, IBSP, and CHRNA9 (Figure 6B). Both two steps showed that compared to cold tumors, antigen-presenting cells and other important immune-related factors are highly infiltrated in hot tumors, which further approve our definition of them (Figures 6A,B).

We conducted Gene Ontology (GO) and Kyoto Encyclopedia of Genes and Genomes (KEGG) enrichment analysis to further confirm the high immune activation level in hot tumors. We found that cytokine–cytokine receptor interaction, allograft rejection, and antigen processing and presentation were enriched in the hot tumor. In the cold tumor, the DEGs were mainly enriched in metabolic pathways, biosynthesis of antibiotics, and neuroactive ligand–receptor interactions. These results suggest that metabolism may influence the immune status of the tumor (Figures 6C–F).

### Identification of Hub Genes for Prognostic and Construction of Predicting Model

**Figure 7:**
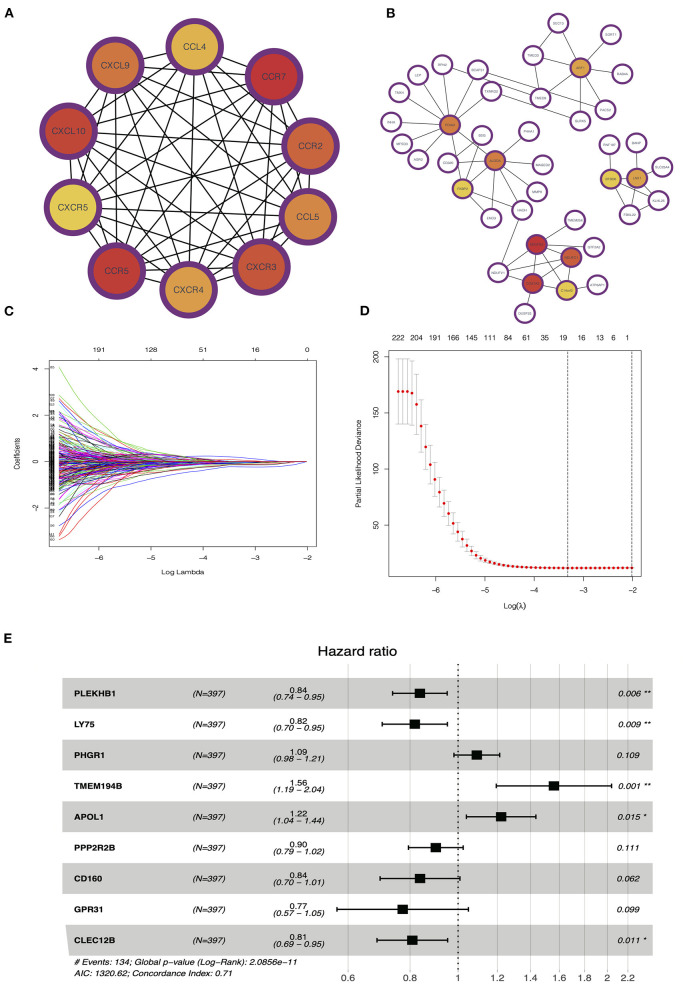
Identification of hub and prognostic-related genes. **(A)** Protein–protein interaction (PPI) network of upregulated genes in the hot tumor. **(B)** PPI network of downregulated genes in the hot tumor. **(C,D)** Least absolute shrinkage and selection operator (LASSO) and partial likelihood deviance coefficient profiles of the selected genes. **(E)** Multivariate Cox analysis showed the hazard ratios (HRs) of selected genes with forest plots.

PPI network analysis was performed to further explore the function of DEGs between hot and cold tumors, and then, we identified hub genes through MCODE in Cytoscape software. The top 10 hub genes in hot tumors mainly regulate the activity of immunocyte chemotactic factors such as CXCL10 and CCL5. The top genes in cold tumor are ARF1, PDIA3, ALDOA, FKBP2, NDUFS5, NDUFC1, COX7A2, C14orf2, LNX1, and BTBD6 (Figures 7A,B).

To evaluate the value of these genes in predicting survival in LUAD, we used the TCGA dataset as a training cohort based on the equal mortality rate. Then, we used a LASSO regression model to identify genes that predict the overall survival in the training cohort. Meanwhile, we also performed a stepwise multi-Cox regression model to identify the genes with the strongest predicting ability. We identified a gene set containing nine genes, in which seven of eight genes were unregulated in the hot tumor, and one of eight was unregulated in the cold tumor; the details in the formation of the nine genes are listed in Figure 7E. We also calculated a risk value as follows: risk value = (−0.1773 × PLEKHB1 expression) + (−0.2011 × LY75 expression) + (0.08690 × PHGR1 expression) + (0.4450 × TMEM194B expression) + (00.1999 × APOL1 expression) + (−0.1034 × PPP2R2B expression) + (−0.1767 × CD160 expression) + (−0.2573 × GPR31 expression) + (−0.2125 × CLEC12B expression). This formula was used to calculate the risk score for each patient in the TCGA and validation cohort (Figures 7C–E).

### Validation of Predicting Model for Overall Survival

**Figure 8:**
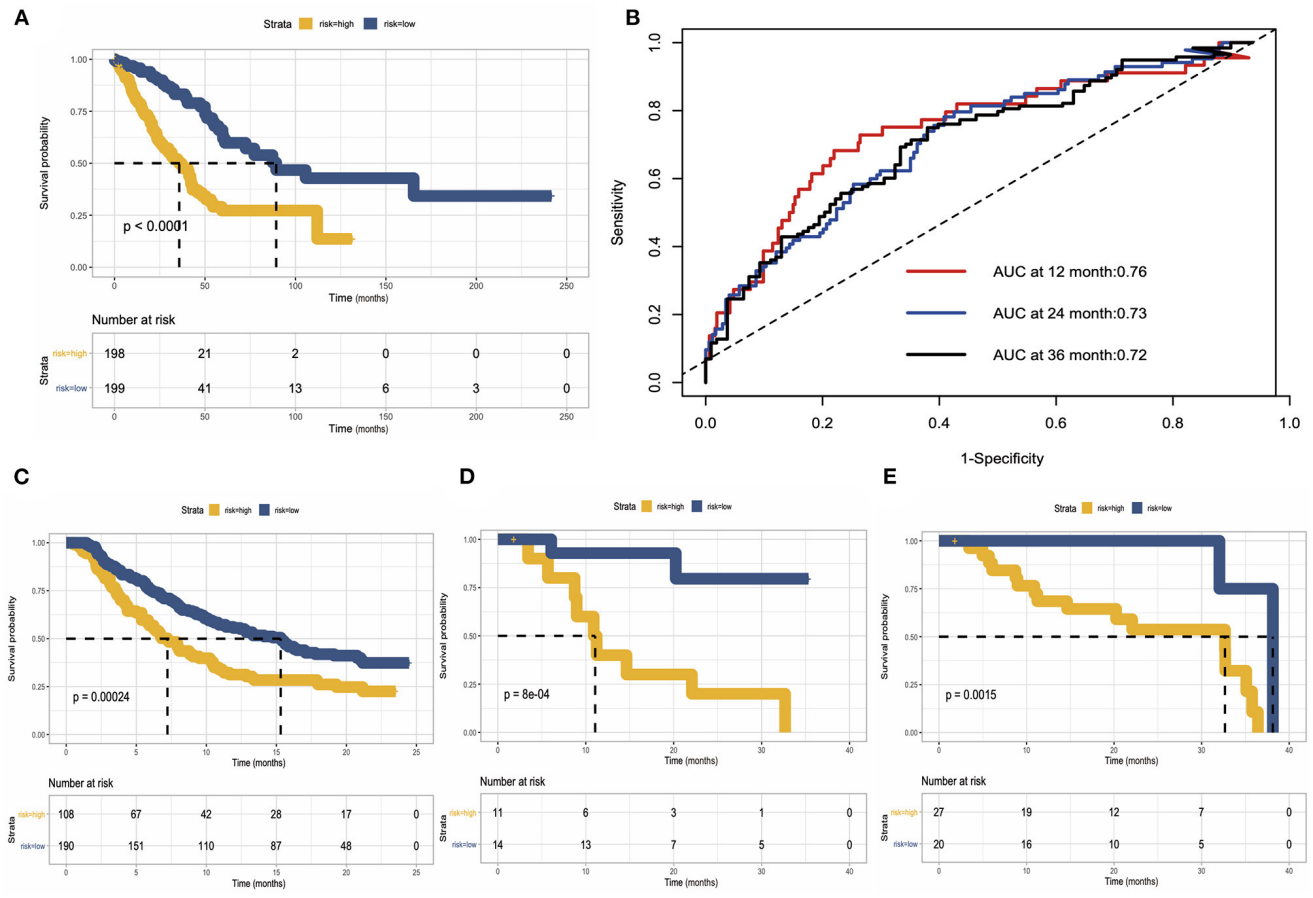
Construction and validation of risk predicting model for overall survival. **(A)** Survival status in the training cohort. **(B)** Receiver operating characteristic (ROC) curve of 1, 2, and 3 years of the training cohort. **(C–E)** Kaplan–Meier survival curve showed the validation of risk predicting model in three external datasets.

As we mentioned before, we use TCGA data as a training cohort, and to further test our model, we used data generated by previous researchers as validation cohort. We built three validation cohorts in total. Survival status showed that the risk score could distinguish the patients well; patients with high-risk score showed unfavorable overall survival in both training and three validation cohort. The areas under the curve (AUCs) of 1, 2, and 3 years for the training cohort were 0.76, 0.73, and 0.72, respectively. These results indicated that the predicting model performed well in predicting overall survival and can be used to guide the clinical management (Figures 8A–E).

## Discussion

Lung cancer is a public health concern for its high morbidity and mortality (Herbst et al., 2018). Due to the high tumor heterogeneity and complex tumorigenic mechanism of lung adenocarcinoma, several challenges exist in developing individual and precise treatment strategies (Fukui et al., 2013); therefore, robust prognosis predicting models is warranted. Accumulating evidence has indicated that prognosis of cancer patients was related to tumor immune infiltration level (Barnes and Amir, 2017; Yang et al., 2019; Zhou et al., 2019), and immune infiltration statuses in tumor microenvironment are key determinants of tumor invasiveness and progression (Gajewski et al., 2013; Ge et al., 2019). With the introduction of immunotherapy, it has been well-established that ICB changed the treatment paradigm of lung cancer, and the application of ICB alone or ICB combined with target therapy/chemotherapy offered hope to many patients who were doomed to death (Karasaki et al., 2017; Mathew et al., 2018). However, there are still patients who are initially or gradually resistant to ICB, and their management is still challenging (Syn et al., 2017). Heterogeneous tumor microenvironment, which is composed of various types of cells that regulated tumor progression, plays an important role in drug resistance (Quail and Joyce, 2013; Wu and Dai, 2017). Therefore, exploring the mechanisms underlying different tumor microenvironment by profiling the cell components are warranted.

In this study, we calculated the immune infiltration of 22 immune cells to comprehensively characterize the functions of these immune cells in the biological process of LUAD. We observed that resting dendritic cells, resting mast cells, and monocytes were positively correlated with both overall survival and progression-free interval of LUAD patients.

The common feature of these cells is that they are involved in antigen presentation process directly or indirectly (Worbs et al., 2017; Murray, 2018; Olivera et al., 2018). While M0 macrophage and follicular helper T cells are associated with poor survival, however, the major function of follicular helper T cells is to help B cells and participate in antibody responses (Crotty, 2019), which seems to be opposite to poor survival. The mechanism behind this phenomenon needs further exploration. Survival results indicate that infiltration status of immune cells can predict patients' survival. In immune-inflamed tumors, we found that several kinds of T cells including CD8^+^ T cells, CD4^+^ T cells, regulatory T cells, and follicular helper T cells are highly infiltrated, consistent with a previous study that T cells are the target of immune checkpoint blocker and Chimeric antigen receptor T cell (CAR-T) therapy, and the status of T cells can exert strong influence on patients' prognosis (Guo et al., 2018). Our results also found that activated immune cells were mainly enriched in tumor tissues, for instance the activated memory CD4^+^ T cell, while naive cells, such as naive B cells, were more abundant in the normal tissue than in tumor tissues.

Synergy and cooperation among different immune cells are essential in the activation of immune response; for example, the process of antigen presentation, recruitment, and stimulation of CD8^+^ T cells are involved with several cell types, chemokines and cytokines (Sánchez-Paulete et al., 2017). We observed that CD8^+^ T cells were correlated with activated memory CD4^+^ T cell, follicular helper T cell, and M1 macrophage cells. However, these correlations were not seen in normal tissues, indicating that immune activation promotes CD8^+^ T cells infiltration. Previous studies have shown that cancer patients of different immune subtypes have distinct prognosis (Denkert et al., 2018; Li et al., 2019; Xu et al., 2020). Based on that, we divided the LUAD into different groups according to the infiltration of 22 immune cells. We identified five clusters with different immune infiltration patterns. Additionally, the immune score, stromal score, and tumor purity of the five clusters are calculated. Immune cell enrichments of clusters 4 and 5 are mainly in several types of T cells, suggesting that this pattern may be more responsive to immunotherapy. Although diversity existed among clusters 1–3, they are similar in general for they shared some features in immune, stromal scores, and tumor purity. Accumulating evidence has indicated that the diversity and density of immune cells in tumor environment play important roles in patients' immune response and prognosis. Based on the status of T cell infiltration and expression of specific cytokine, the tumor microenvironment can be simply defined into hot and cold tumors. In our study, we redefined clusters 4 and 5 as hot tumor group and others as cold tumor group. Results showed that the two types of tumors behaved differently at the transcriptome level. Consistent with the high immune score observed in hot tumors, immune-related genes were highly expressed in hot tumors, including CXCL9, TCL1A, CCL19, CXCL13, MS4A1, and C4orf7. Matrix metallopeptidase 8 (MMP8) is one of the highly expressed genes in cold tumors, and it encodes a member of the matrix metalloproteinase (MMP) family, which is involved in the breakdown of extracellular matrix including extracellular molecules and a number of bioactive molecules (Juurikka et al., 2019). Go annotations related to this gene include metalloendopeptidase activity. It has been reported that MMP8 behaved differently in cancers depending on their tissue of origin and was a potential prognostic factor (Juurikka et al., 2019). In lung cancer, MMP8 is believed to be associated with a decreased lung cancer risk, and its profile was distinctly different according to histological types and patient recurrence status (Shah et al., 2010). The function of MMP8 in cold tumors needs further exploration. GO and KEGG enrichment analysis revealed that hot tumor was enriched in cytokine–cytokine receptor interaction and antigen processing and presentation. As to cold tumors, the DEGs were mainly enriched in metabolic pathways; metabolic dysfunction is the mechanism behind many malignant behaviors of tumor (Chen et al., 2019).

Hub genes in hot tumors mainly were involved with immunocyte chemotaxis, such as chemotactic factors CXCL10 and CCL5; however, it was difficult to link most hub genes in cold tumors with immune activities. Previous studies have evaluated the ability of immune cells in predicting prognosis of cancer (Gentles et al., 2015; Shen et al., 2019), and based on that, we explored the prognostic value of DEGs in our study. We developed a risk model containing nine genes. After detailed exploration of the nine genes, we identified three genes, APOL1, CD160, and PPP2R2B, for further study. Apolipoprotein L1 (APOL1) is a protein-coding gene that is associated with focal segmental glomerulosclerosis and glomerulonephritis. It is correlated with lipid binding and chloride channel activity, and in our model, it is associated with unfavorable prognosis. Previous studies have reported that the AOPL1 is a protective factor for renal carcinoma (Hu et al., 2012), but the function of APOL1 in LUAD has not been fully illustrated. CD160 molecule (CD160) is a protein-coding gene associated with neurotrophic keratopathy and cone-rod dystrophy 1. It has been reported that CD160 is expressed on activated NK or T cells in humans and regulated the cytokine production of NK cells, therefore regulating its function (Tu et al., 2015). It has also been reported that CD160 is involved in T-cell regulation in immune response of the virus (Cai and Freeman, 2009). GO annotation results suggest that CD160 is related to innate immunity. In our model, CD160 is associated with a better prognosis. Protein phosphatase 2 regulatory subunit B beta (PPP2R2B) is a protein-coding gene associated with diseases including spinocerebellar ataxia, and in our study, it is associated with favorable survival. It has been reported that, in colorectal cancer, PPP2R2B, encoding the B55β regulatory subunit of the PP2A complex, is epigenetically inactivated by DNA hypermethylation and is related to the rapamycin sensitization (Tan et al., 2010). The roles of PPP2R2B in lung cancer need further exploration.

We used the TCGA dataset as a training cohort, and our risk-predicting model showed satisfying efficacy in external datasets. Three credible datasets were chosen as external validation, which was a large phase 2 trial (IMvigor210) investigating the clinical activity of atezolizumab in metastatic urothelial cancer (Mariathasan et al., 2018), 38 pretreatment (pembrolizumab and nivolumab) melanoma tumors (Hugo et al., 2016), and 68 patients with advanced melanoma (CA209-038 study) (Riaz et al., 2017). Although our predicting model was constructed based on the LUAD data, it behaved well in other cancer types (urothelial cancer and malignant melanoma) and other datasets, which further indicated the stability and reliability of our model, and implied the potentiality that our model could be utilized in more cancer types. In conclusion, we constructed a risk prediction model using immune cell infiltration status. Since it is the era of immune therapy and lung cancer is one of the most malignant cancer in the world, it is reasonable and prompt to construct risk prediction model using immune-related information. Our model can spot patients with high risk in immunotherapy resistance accurately and therefore may guide the clinical use of immune therapy.

## Data Availability Statement

The original contributions presented in the study are included in the article/supplementary material, further inquiries can be directed to the corresponding author/s.

## Author Contributions

JH and CL conceived and designed the study. YZ, HT, HL, CX, and ZZ conducted experiments and data analysis. YL, LW, TF, GG, FT, QX, and BZ performed data collection and analysis. CL, YZ, and HT wrote the manuscript. All the authors approved the manuscript.

## Conflict of Interest

The authors declare that the research was conducted in the absence of any commercial or financial relationships that could be construed as a potential conflict of interest.
